# Seroprevalence of Antibodies against Seal Influenza A(H10N7) Virus in Harbor Seals and Gray Seals from the Netherlands

**DOI:** 10.1371/journal.pone.0144899

**Published:** 2015-12-14

**Authors:** Rogier Bodewes, Ana Rubio García, Sophie M. Brasseur, Guillermo J. Sanchez Conteras, Marco W. G. van de Bildt, Marion P. G. Koopmans, Albert D. M. E. Osterhaus, Thijs Kuiken

**Affiliations:** 1 Department of Viroscience, Erasmus MC, Rotterdam, the Netherlands; 2 Seal Rehabilitation and Research Centre, Pieterburen, the Netherlands; 3 IMARES—Institute for Marine Resources & Ecosystem Studies, Wageningen University, Texel, the Netherlands; 4 National Institute of Public Health and the Environment, Centre for Infectious Disease Control, Bilthoven, the Netherlands; 5 Artemis One Health, Utrecht, the Netherlands; 6 Research Centre for Emerging Infections and Zoonoses, University of Veterinary Medicine Hannover, Hannover, Germany; Sonoma State University, UNITED STATES

## Abstract

In the spring and summer 2014, an outbreak of seal influenza A(H10N7) virus infection occurred among harbor seals (*Phoca vitulina*) off the coasts of Sweden and Denmark. This virus subsequently spread to harbor seals off the coasts of Germany and the Netherlands. While thousands of seals were reported dead in Sweden, Denmark and Germany, only a limited number of seals were found dead in the Netherlands. To determine the extent of exposure of seals in the Netherlands to influenza A/H10N7 virus, we measured specific antibody titers in serum samples from live-captured seals and seals admitted for rehabilitation in the Netherlands by use of a hemagglutination inhibition assay and an ELISA. In harbor seals in 2015, antibodies against seal influenza A(H10N7) virus were detected in 41% (32 out of 78) pups, 10% (5 out of 52) weaners, and 58% (7 out of 12) subadults or adults. In gray seals (*Halichoerus grypus*) in 2015, specific antibodies were not found in the pups (n = 26), but in 26% (5 out of 19) of the older animals. These findings indicate that, despite apparent low mortality, infection with seal influenza A(H10N7) virus was geographically widespread and also occurred in grey seals.

## Introduction

In the past few decades, various outbreaks of mortality among harbor seals (*Phoca vitulina*) caused by influenza A viruses have been reported along the east coast of North America [1,2,3,4], but not in European waters. In addition, serological studies suggest that harbor seals are exposed to influenza A viruses of multiple subtypes (for review see: [1]). Phylogenetic analyses of the influenza A viruses isolated from harbor seals indicated that viruses detected during major outbreaks were most closely related to influenza A viruses circulating among birds [1,2,3,4]. Furthermore, it has been demonstrated that seals are susceptible to infection with human influenza viruses, e.g. the pandemic influenza A(H1N1)2009 virus was detected in northern elephant seals (*Mirounga angustirostris*) and influenza B viruses were detected in harbor and gray seals (*Halichoerus grypus*) [5,6,7].

In spring and summer 2014, increased mortality was reported among harbor seals along the coasts of Sweden and Denmark, associated with infection by an influenza A(H10N7) virus [8]. Genetic analysis of the influenza A(H10N7) virus detected in seals indicated that this virus was most closely related to avian influenza A(H10N7) viruses from wild birds [8,9,10]. In the autumn of 2014, the seal influenza A(H10N7) virus spread to seals along the coast of Germany, which resulted in the death of between 1500 and 2000 seals [9] and the virus was also detected in dead seals along the coast of the Netherlands from early November 2014 until early January 2015.

Of interest, while thousands of dead seals were reported along the coast of Germany, only a very limited number of harbor seals (<180) were found dead along the coast of the Netherlands. This raised the question whether the seal influenza A(H10N7) virus had indeed continued to spread among the harbor seals along the Dutch coast or that spread was limited. Main factors that could have limited the spread of the virus include differences in herd immunity and differences in timing of the virus arrival, related to the seasonal behavior of the seals off the coast of the Netherlands (e.g. less contact between harbor seals in the autumn and winter seasons). In addition, genetic changes in the virus could have resulted in a lower virulence of the virus for harbor seals, resulting in less severe disease following infection. However, it might also be possible that infection and/or deaths did occur but that the south to east wind direction that occurred in November 2014 in the Netherlands [11] resulted in less stranded seals by blowing carcasses towards the North Sea, as was observed during the outbreak of phocine distemper virus (PDV) in 2002 [12].

In the present study, the spread of seal influenza A(H10N7) virus among seals of the Dutch coastal waters was evaluated by assessing the seroprevalence of antibodies against the seal influenza A(H10N7) virus in serum samples collected from harbor seals and gray seals.

## Materials and Methods

### Ethics statement

Serum samples of seals used in the present study were obtained by the Seal Research and Rehabilitation Centre (SRRC), Pieterburen, the Netherlands and by IMARES—Institute for Marine Resources & Ecosystem Studies, Wageningen University, the Netherlands, and the SRRC and IMARES provided permission to the Department of Viroscience, Erasmus Medical Centre to use the serum samples for the present study. Admission and rehabilitation of wild seals at the SRRC is permitted by the government of the Netherlands (application number FF/75/2012/015) and serum samples provided by the SRRC were collected from blood samples that were collected for routine diagnostics during rehabilitation. Serum samples provided by IMARES were collected from live-captured (and subsequently released) seals in frame of various ecological studies which was permitted by the government of the Netherlands (application number FF/75A/2013/22). Blood sampling was approved by the independent Ethical Committee for Animal Experiments of the Royal Netherlands Academy of Arts and Sciences. Capturing of live seals was performed at the following locations/areas off the coast of the Netherlands: Aardappelbult (Zeeland), Blauwe Balg (Ameland), Dollart (Eems), Hond/Paap (Eems), Pinkegat (Ameland), Renesse (Zeeland), Sparregat (Eems), Steenplaat (Texel) (S1 Fig).

### Serum collection

Blood samples used were collected from live-stranded harbor and gray seals that were admitted to the SRRC for rehabilitation from 2010 to 2015. Blood samples were collected for diagnostic purposes and remaining serum was stored at -20°Cuntil analysis. In addition, blood samples were collected from live-captured and subsequently released harbor and gray seals in 2010, 2011, 2012, 2014 and 2015 by IMARES. The age of the rehabilitated seals was estimated based on the date of stranding and the body size. Harbor seals were divided into three age categories; harbor seal pups (<1 month of age at the day of blood collection; stranded between May and July; length nose-tail 70–90 cm; umbilical cord present/freshly absent), harbor seal weaners (between one month and one year of age; stranded between August and April; maximum length nose-tail 110 cm) and harbor seal (sub)adults (older than one year of age; length nose-tail >105–110 cm). Since the estimation of the ages of seals older than one year of age was unclear, subadult and adult seals were not differentiated; these two categories were referred together as “(sub)adults” in the rest of the text. Gray seals were divided into two groups; gray seal pups (less than one month of age; stranded between November and January; length nose-tail 95–105 cm; white coat; umbilical cord present/freshly absent) and non-pup older gray seals (older than 2–3 months of age; absence of white coat; length nose-tail >105 cm). The ages of live-captured harbor and grey seals were estimated based on the length from nose to tail as indicated for stranded seals. Since the length from nose to tail of each live-captured seal was at least 120 cm, all animals were estimated to be (sub)adults. A summary of the serum samples tested is provided in Table 1.

**Table 1:** Species and age category of seals from which serum samples were tested.

	Admitted for rehabilitation (SRRC)															Live-captured (IMARES)					
	Harbor seals									Gray seals						Harbor seals			Gray seals		
Year	Pup			Weaner			(Sub)adult			Pup			Non-pup			(Sub)adult			Non-pup		
	T^1^	NP^2^	HI^3^	T	NP	HI	T	NP	HI	T	NP	HI	T	NP	HI	T	NP	HI	T	NP	HI
2010	2	0	0	15	0	0	0	0	0	1	0	0	3	0	0	18	0	0	0	0	0
2011	16	0	0	33	0	0	1	0	0	0	0	0	2	0	0	29	1	1	0	0	0
2012	0	0	0	21	0	0	0	0	0	2	0	0	0	0	0	0	0	0	0	0	0
2013	25	0	0	11	0	0	5	0	0	7	0	0	1	0	0	9	0	1	16	0	1
2014	19	0	0	34	0	0	1	0	0	22	2	1	3	0	0	24	0	0	14	0	1
2015	78	12	32	52	5	8	0	0	0	26	0	0	3	0	0	12	7	7	19	1	5

^1^: number of animals tested

^2^: number of animals tested positive in the NP Elisa

^3^: number of animals tested positive in the HI assay

### Serology

All collected serum samples were tested using an influenza A virus nucleoprotein (NP) blocking ELISA (IDEXX Influenza A Ab Test) according to the instructions of the manufacturer. This assay detects antibodies against any of the influenza A virus subtypes. A value of sample to negative control ratio of <0.6 was considered positive. In addition, serum samples collected from all (sub)adult harbor seals and non-pup gray seals and the serum samples collected from pups and weaners in 2014 and 2015 were tested for the presence of antibodies against the hemagglutinin (HA) of seal influenza A(H10N7) virus using the hemagglutination inhibition (HI) assay following a standard protocol using 1% turkey erythrocytes and four HA-units of virus [13]. A reverse genetics virus containing the HA of influenza A/harbor seal/PV14-221_TS/NL and the remaining gene segments of influenza A/PR/8/34 was produced as described previously and used as antigen in the HI assay [14]. An HI titer of 1:20 or higher was considered positive. In both assays, serum samples from influenza A(H10N7) infected and uninfected ferrets were used as positive and negative controls, respectively.

## Results

### Detection of antibodies against the influenza A virus NP

**Fig 1:**
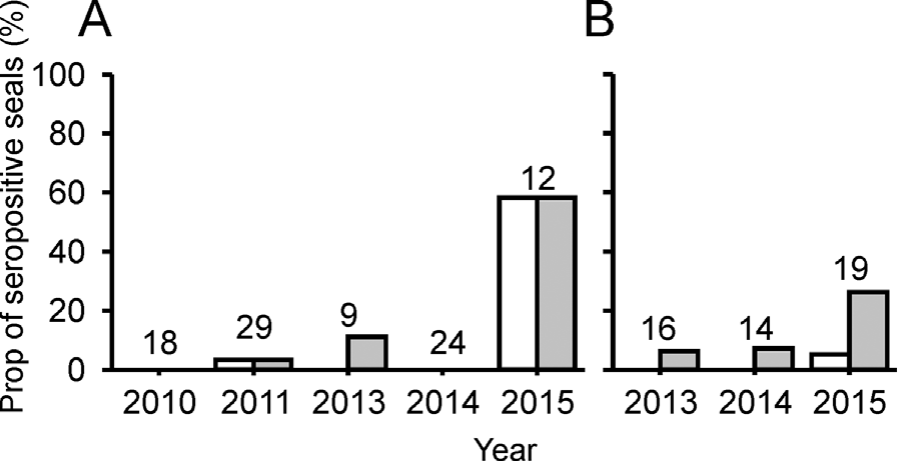
Proportion of live-captured seals with antibodies against influenza A virus. Serum samples collected from live-captured (sub)adult harbor (**A**) and gray (**B**) seals were tested for the presence of antibodies against the NP using an influenza A virus NP blocking ELISA (white bars) and the hemagglutination inhibition test (gray bars). The numbers above the bars indicate number of sera tested.

**Fig 2:**
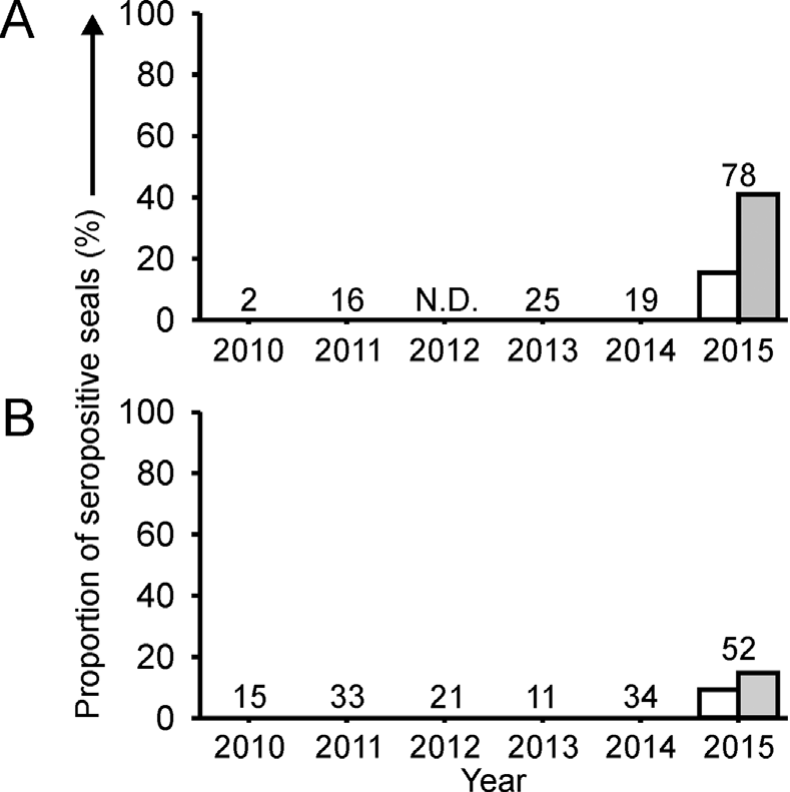
Proportion of seals admitted to the SRRC with antibodies against influenza A virus. Serum samples collected from harbor seal pups (**A**) and harbor seal weaners (**B**) seals were tested for the presence of antibodies against the NP using an influenza A virus NP blocking ELISA (white bars) and the hemagglutination inhibition test (gray bars). N.D.: no data (see Table 1). The numbers above the bars indicate number of sera tested.

In serum samples collected from 2010 to 2013, antibodies against the influenza A virus NP were detected in 1 (a [sub]adult harbor seal) out of 72 serum samples (1%). In 2014, NP-specific antibodies were detected in 2 out of 22 gray seal pups (9%) from which serum was collected in December of that year. In serum samples collected from harbor seals in 2015, NP-specific antibodies were detected in 12 out of 78 stranded pups (15%), 5 out of 52 stranded weaners (10%) and 7 out of 12 live-captured (sub)adult seals (58%). In serum samples collected from gray seals in 2015, NP-specific antibodies were only detected in 1 out of 19 (5%) non-pup live-captured gray seals (Figs 1 and 2).

### Detection of antibodies against seal influenza A(H10N7) virus

Using the HI assay, serum samples collected from all (sub)adult seals were tested for antibodies against seal influenza A(H10N7) virus. From 2010 to 2013, antibodies were detected in 1 out of 29 (sub)adult harbor seals in 2011 (3%), 1 out of 9 (sub)adult harbor seals in 2013 (11%) and 1 out of 16 (6%) non-pup gray seals in 2013. In serum samples collected in 2014, antibodies were detected in 1 out of 14 (7%) non-pup live-captured gray seals in 2014. In 2015, antibodies were detected in 7 out of 12 (58%) (sub)adult live-captured harbor seals and in 5 out of 19 (26%) non-pup live-captured gray seals.

**Fig 3:**
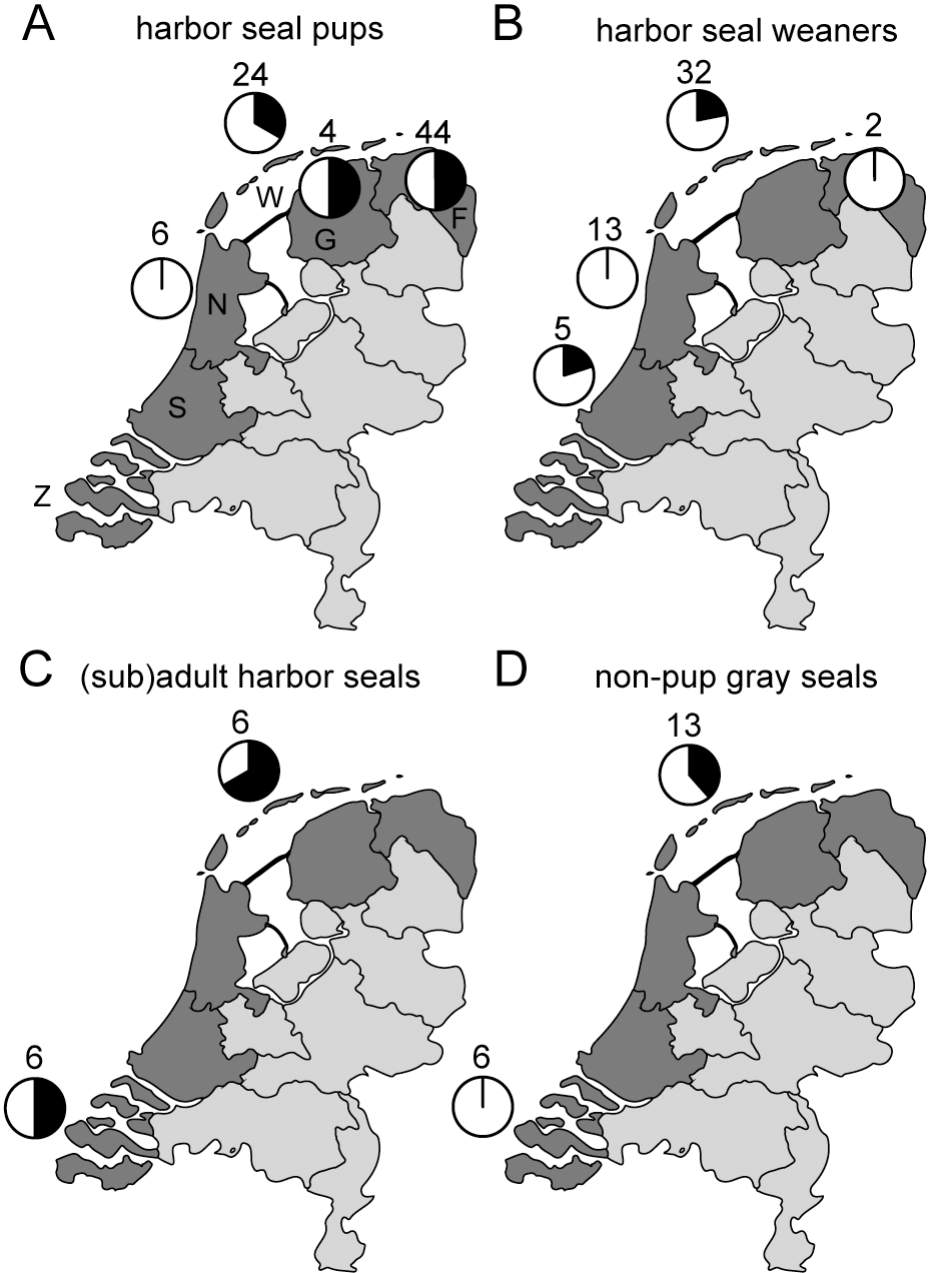
Geographical distribution of seals with H10N7 antibodies in 2015 in the Netherlands. Map of the Netherlands with proportion (black sector) of the total (complete pie) harbor seal pups (A), harbor seal weaners (B), (sub)adult harbor seals (C) and non-pup gray seals (D) with antibodies against the seal HA of influenza A(H10N7) virus. The coastal regions where samples were collected in 2015 are indicated in dark grey: (Wadden Islands, W; Groningen, G; Friesland, F; North-Holland, N; South-Holland, S; and Zeeland, Z). The number above the pie charts indicate the number of serum samples tested per region. The map of the Netherlands was adapted from: https://pixabay.com/en/netherlands-holland-map-europe-303419/.

Serum samples also were tested of pups and weaners that were admitted for rehabilitation to the SRRC. From 2010 to 2013, no antibodies against influenza A(H10N7) virus were detected in any harbor seal pups (n = 43), harbor seal weaners (n = 80), gray seal pups (n = 10), or non-pup gray seals (n = 16). In 2014, antibodies were detected in 1 out of 22 gray seal pups (5%), but not in any harbor seal pups (n = 19) or harbor seal weaners (n = 34). The seropositive gray seal pup had been admitted to the SRRC in December 2014. In 2015, antibodies against influenza A(H10N7) were detected in 32 out of 78 harbor seal pups (41%) and in 8 out of 52 harbor seal weaners (15%), but not in any gray seal pups (n = 26) or non-pup gray seal (n = 3) (Figs 1 and 2). To elucidate whether the seal A(H10N7) virus had spread all along the Dutch coast, the locations of stranding or capture of the seals were evaluated. Antibodies were detected in seals in all coastal regions of the Netherlands (Fig 3).

### HI antibody titers and duration of antibody response

**Fig 4:**
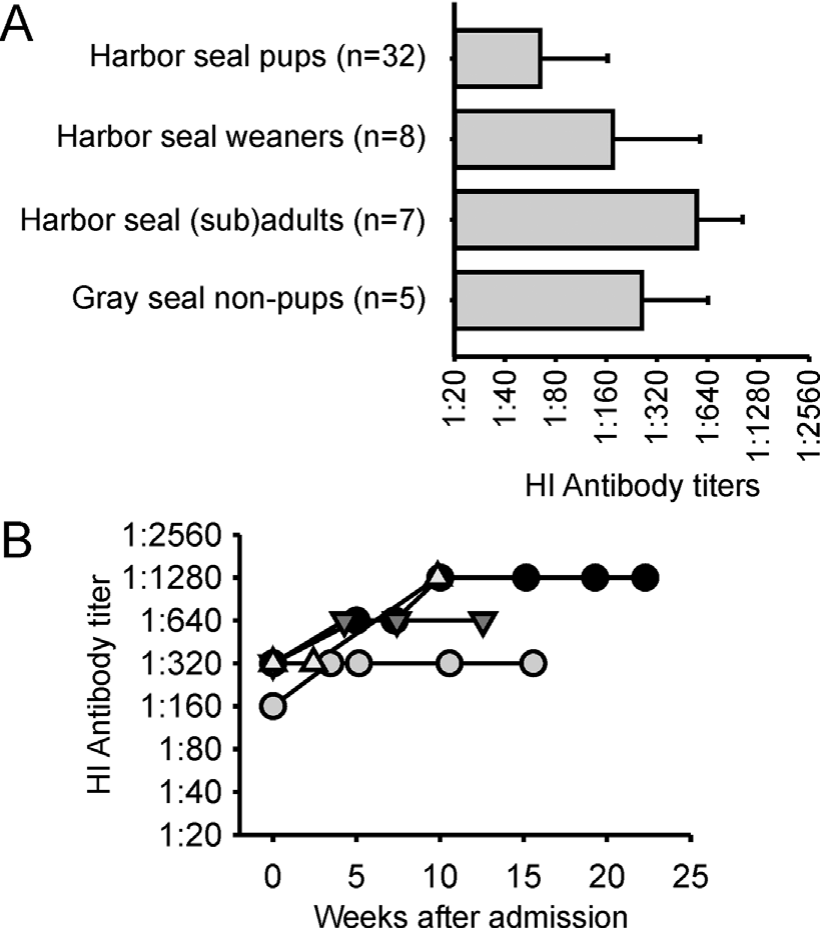
Geometric mean antibody titers and duration of antibodies against influenza A(H10N7). GMT (± s.d.) of seals with antibodies against influenza A(H10N7) virus in 2015 (**A**). The numbers next to the group names indicate the number of sera tested. Temporal change in HI antibody titers in four individual harbor seal weaners that tested seropositive upon admission (**B**).

Seal influenza A(H10N7) virus-specific antibody titers detected in (sub)adult seals before the start of the outbreak in 2014 ranged between 1:40 and 1:80 (n = 3), while the geometric mean titers (GMT) of antibodies detected in (sub)adult harbor seals in 2015 against the A/harbor seal/PV14-221_TS/NL strain was 1:573 (s.d. 89) (n = 7) and GMT of antibodies detected in non-pup gray seals was 1:272 (s.d. 75) (n = 5). The single gray seal pup with antibodies against H10N7 that was admitted in 2014 had an antibody titer of 320, while the GMT in harbor seal pups was 1:68 (s.d. 34) (n = 32) and the GMT in harbor seal weaners was 1:180 (s.d. 75) (n = 8) (Fig 3). To study the duration of the antibody response against seal influenza A/H10N7 virus, four harbor seal weaners were identified that tested positive in the first serum sample collected upon admission. From these four seals, serum samples that had been collected subsequently for diagnostic purposes between 1 and 156 days after admission were tested for antibodies against the seal influenza A(H10N7) virus. NP antibodies were detected in all serum samples and the HI antibody titers in these serum samples did not decrease with time (Fig 4).

## Discussion

To study the spread of the seal influenza A(H10N7) virus among harbor and gray seals of the Dutch coastal waters, the seroprevalence of antibodies against seal influenza A(H10N7) viruses in serum samples collected from various age groups of seals off the Netherlands was evaluated using a NP blocking ELISA and the HI assay. Although serum samples from a relatively low number of samples was tested compared to the population of harbor seals, the results of this study indicate that the seal influenza A(H10N7) virus had spread among the harbor seals off the coast of the Netherlands in 2014/2015. This conclusion is supported by the prevalence of 58% HI antibodies against seal influenza A(H10N7) virus in (sub)adult harbor seals captured in 2015, compared to 11% or less in preceding years (Fig 1). Also, HI antibodies against seal influenza A(H10N7) virus in rescued harbor seal pups and weaners were found only in 2015 (41% and 15% prevalence, respectively), and not in preceding years (Fig 2). The presence of antibodies in seal pups with a relatively low antibody titer is most likely explained by transfer of maternal antibodies as has been previously observed in harbor seals with antibodies to PDV [15,16]. Besides the relatively high number of harbor seals with serum antibodies against seal influenza A(H10N7) virus in 2015, a small number of (sub)adult harbor and gray seals sampled before 2015 had low antibody titers against this virus. The presence of low antibody titers against the seal influenza A/H10 virus in the (sub)adult seals might be explained by infection some time ago or infection with an influenza A virus of the same subtype (H10), yet antigenically distinct from this seal influenza A(H10N7) virus. The low seroprevalence before the outbreak may indicate that no herd immunity existed at the start of the outbreak and that the relatively low mortality of the seals off the coast of the Netherlands could be due to the seasonal behavior of the seals or genetic changes in the virus that resulted in a variant that was less virulent for harbor seals. Therefore, there is the possibility that the seal influenza A(H10N7) virus might also have spread to other harbor seal populations in the region, such as off the coasts of France and the United Kingdom, without causing increased numbers of stranded or dead seals.

Of interest, one gray seal pup tested negative in the HI assay and positive in the NP ELISA. This might be explained by infection with an influenza A virus of another subtype. Also, 29 harbor seals, in which antibodies were detected using the influenza A/H10-specific HI assay, tested negative in the NP ELISA. This might be explained by differences between the two assays and differences in the protein of the influenza A virus to which the assay was directed. While the influenza A virus NP blocking ELISA detects antibodies against all influenza A virus subtypes, the HI assay is specific for detecting antibodies against influenza A(H10N7) virus.

To our knowledge, no seal influenza A(H10N7) virus was detected in gray seals during the recent influenza A(H10N7) outbreak and no increased mortality of gray seals has been reported in northwestern Europe. The detection of antibodies against the seal influenza A(H10N7) virus in 26% of live-captured (sub)adult gray seals indicates that they are susceptible to infection with the seal influenza A(H10N7) virus, but that infection may not result in morbidity or mortality. Antibodies against influenza A viruses in gray seals have been reported previously [17]. The difference in reported morbidity and mortality between harbor and gray seals as seen with infection with seal influenza A(H10N7) virus is similar to that following PDV infection, which caused massive mortality in harbor seals in 2002 in northwestern Europe, but not in gray seals [12]. We cannot explain why gray seals are apparently more resistant to disease from these two virus infections than harbor seals.

In conclusion, in the present study we provide serological evidence that the seal influenza A(H10N7) virus spread among harbor seals off the Dutch coast in 2015, as well as among gray seals. In addition, because the majority of harbor seal pups (59%) and weaners (85%) in 2015 had no detectable antibodies to seal influenza A(H10N7) virus, continued circulation of this virus after the recent outbreak seems unlikely. Continued monitoring and additional studies are necessary to elucidate the spread of the influenza A(H10N7) virus among the various populations of seals of Northwestern Europe.

## Supporting Information

S1 Fig Overview of locations were seals were captured (and released) off the coast of the Netherlands by IMARES. Capturing of live seals was performed at the following locations/areas off the coast of the Netherlands: A: Aardappelbult (Zeeland), B: Blauwe Balg (Ameland), D: Dollart (Eems), HP: Hond/Paap (Eems), P: Pinkegat (Ameland), R: Renesse (Zeeland), Sp: Sparregat (Eems), St: Steenplaat (Texel).
(TIF)
