# Supplementary figures and images for: Seroprevalence of Antibodies against Seal Influenza A(H10N7) Virus in Harbor Seals and Gray Seals from the Netherlands
Source: PLoS One. 2015 Dec 14;10(12):e0144899. doi: 10.1371/journal.pone.0144899 (PMC4684379; doi:10.1371/journal.pone.0144899)

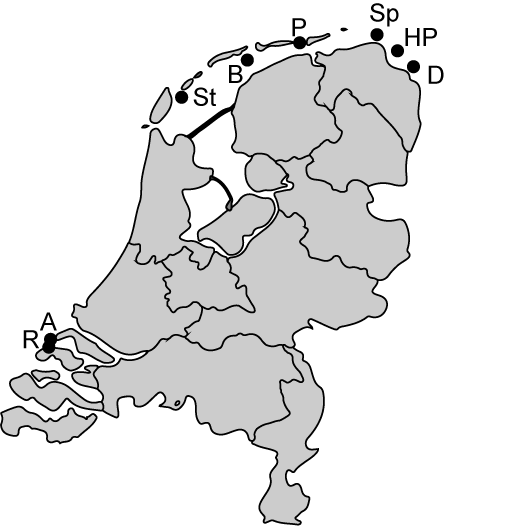

Supplement: S1 Fig — Capturing of live seals was performed at the following locations/areas off the coast of the Netherlands: A: Aardappelbult (Zeeland), B: Blauwe Balg (Ameland), D: Dollart (Eems), HP: Hond/Paap (Eems), P: Pinkegat (Ameland), R: Renesse (Zeeland), Sp: Sparregat (Eems), St: Steenplaat (Texel). (TIF) [file pone.0144899.s001.tif]
